# Evaluation of Morphometric Findings of Corpus Callosum in Schizophrenia Patients with Magnetic Resonance Imaging and Comparison with Healthy Individuals

**DOI:** 10.3390/jcm14061961

**Published:** 2025-03-14

**Authors:** Hasan Hüsnü Yüksek, Seda Türkili, Ayten Yüksek, Barış Ten, Şadiye Visal Buturak

**Affiliations:** 1 Department of Radiology, Faculty of Medicine, Mersin University, 33110 Mersin, Türkiye; 2 Department of Psychiatry, Faculty of Medicine, Mersin University, 33110 Mersin, Türkiye; 3 Department of Psychiatry, Mersin City Education and Research Hospital, 33240 Mersin, Türkiye

**Keywords:** schizophrenia, corpus callosum, magnetic resonance imaging, morphometry, index

## Abstract

**Objective:** We aimed to compare the forebrain length, corpus callosum sub-segment thickness, corpus callosum area, and corpus callosum index in the cerebral magnetic resonance imaging (MRI) of schizophrenia patients and healthy individuals. **Materials and Methods:** In this retrospective study, 137 schizophrenia patients who met the inclusion and exclusion criteria and were hospitalized in the Psychiatry Clinic of Mersin University Faculty of Medicine Hospital between January 2014 and January 2024 and 137 healthy individuals of the same age and gender without any mental disorders were included. The relationship between sociodemographic characteristics and disease-related variables obtained in the retrospective file review and the corpus callosum morphometric findings on brain MRI were analyzed with the SPSS 22 package program. *p*-values below 0.05 were considered statistically significant. **Results:** In the study, 274 individuals, including 137 schizophrenia patients (59 [43.1%] males; 78 [56.9%] females) and 137 healthy individuals (59 [43.1%] males; 78 [56.9%] females), were evaluated. There was no significant difference between the two groups in terms of age, gender, and mean age at the time of brain MRI. In brain MRI measurements, forebrain length, corpus callosum (CC) AP diameter, CC genu, body, splenium, rostrum and isthmus thicknesses, CC area, and CC index values were significantly lower in the patient group compared to healthy controls. It was also found that patients with multiple episodes in the patient group were diagnosed at an earlier age, had a longer duration of illness, had a history of more homicide and suicide attempts, had more hospitalizations, had a history of more psychotic disorders in their families, and had lower levels of functioning compared to patients with a single episode. **Conclusions:** Each of the corpus callosum subregion thickness, corpus callosum area, and corpus callosum index values shows a decrease in schizophrenia patients compared to healthy controls. These findings contribute to the understanding of the neurobiological basis of the disease and provide important evidence to elucidate its pathophysiology. The results need to be confirmed in studies with larger samples using a prospective study design in which clinical parameters related to the disease are also measured.

## 1. Introduction

Schizophrenia is a serious psychiatric disorder that usually starts at a young age, whose course and outcomes vary from individual to individual and affects all aspects of the mental state [1]. The World Health Organization (WHO) defines schizophrenia as one of the top 10 diseases that contributes most to the global burden of disease [2]. This chronic condition, which has a complex and heterogeneous structure, leads to a significant loss of function in individuals and creates serious social and economic burdens on families, society, and health systems [3,4]. The diagnosis of schizophrenia is based on subjective methods such as clinical history taken from the patient and his/her relatives, mental status examination, and observed clinical symptoms.

Although the etiopathogenesis of schizophrenia is still not fully known, it is accepted that it represents a cluster of syndromes consisting of various diseases with similar signs and symptoms [5]. In addition to various genetic factors, obstetric complications, infections, changing inflammatory processes, substance abuse, differences observed in brain morphology, and neurotransmitter dysfunctions are involved in the etiology of the disease [6,7,8]. The wide range of clinical symptoms seen in schizophrenia and the diversity of impairment in neurocognitive functions suggest that more than one brain region is involved in the development of the disease. In this context, many brain regions have been evaluated; an important one of them has been the corpus callosum and its subregions.

The corpus callosum is the brain’s largest and most functionally important structure that provides interhemispheric connectivity. It has a critical role in providing high cognitive functions between the brain’s two hemispheres and in the transfer of sensory and motor information [9,10]. It has been reported that various neurologic and psychiatric disorders may be related to morphological and functional differences in the corpus callosum. In schizophrenia patients, the neurobiological basis of the changes observed in this structure and its relationship with clinical symptoms play a key role in understanding the pathophysiology of the disease.

Although the corpus callosum has no anatomically distinct border, it is topographically divided into five subdivisions from anterior to posterior as follows: rostrum, genu, body, isthmus, and splenium [11,12]. Fiber structures characterize each subregion and connect different cortical regions. The anterior parts of the corpus callosum connect bilateral prefrontal cortices. The posterior parts of the corpus callosum connect the temporo-parietal regions and are mainly associated with visual and language processing. The body is the subregion of the corpus callosum responsible for sensory–motor information transfer [13,14,15].

Studies on callosal length and thickness have reported different results. Differences between studies may be due to the lack of corrections for age, gender, brain size, patient groups, or differences in the devices and measurement methods used. Various researchers have proposed measurements such as the corpus callosum area and corpus callosum index in this context. Corpus callosum index measurement offers the opportunity to make individualized brain size corrections and provides more accurate and consistent personal measurements in the follow-up of the same individual [16].

Various studies have shown inter-hemispheric and intra-hemispheric connection disorders in schizophrenia patients. In this context, it has been suggested that the corpus callosum is an important structure in schizophrenia [15,17]. Studies have shown consistent findings with decreases in white matter density in all and/or subregions of the corpus callosum in schizophrenia patients [18,19,20,21]. Similarly, post-mortem studies have reported a significant decrease in fiber density in the corpus callosum of individuals diagnosed with schizophrenia [22].

With the recent rapid development and progress in brain imaging methods, these methods are frequently used to elucidate the etiopathogenesis of psychiatric disorders. Brain magnetic resonance imaging is an effective and non-invasive method that can be used in this context. A better understanding of the etiology and risk factors of schizophrenia will enable the identification of groups at risk and the development of new treatment approaches. In addition, early recognition of the disease will be possible in this way, and complications that may cause disability can be prevented.

Our study aimed to compare the forebrain length, corpus callosum sub-segment thicknesses, corpus callosum area, and corpus callosum index in the cerebral magnetic resonance imaging of patients with schizophrenia and healthy control subjects.

## 2. Materials and Methods

The study was conducted as a single-center retrospective written and electronic archive file review. Approval for the study was obtained from the Mersin University Clinical Research Ethics Committee, with the board decision dated 2 October 2024 and numbered 2024/917.

### 2.1. Study Participants

In the study, a patient group was formed with 137 cases by considering the exclusion criteria among 477 adult patients aged 18 years and older who were treated as inpatients in the psychiatry clinic of Mersin University Faculty of Medicine Hospital between January 2014 and January 2024. Participants in the patient group were diagnosed with schizophrenia according to DSM-5 diagnostic criteria as a result of a mental status evaluation by a psychiatrist. A healthy control group was formed with 137 individuals of the same age and gender as the individuals in the patient group. This group was randomly selected from outpatients who applied to non-psychiatric medical specialty departments of the hospital and had nonspecific symptoms based on information obtained from their medical records. The selection was made by considering the exclusion criteria for the healthy control group.

The exclusion criteria for the groups were as follows:

Patient group:

1. Alcohol or substance use disorder, bipolar affective disorder, gestational and related psychoses, and other mental disorders (anxiety and related disorders, depressive disorders, etc.).
2. Psychoses associated with central nervous system pathologies.
3. Intellectual disabilities and/or pervasive developmental disorders.
4. History of oncologic disease.
5. History of previous intracranial surgery and imaging findings.
6. No evaluable magnetic resonance imaging study.

Healthy control group:

1. Past and current psychiatric admission and any psychiatric diagnosis.
2. History of previous intracranial surgery and imaging findings.
3. Organic pathology, major intracranial anomaly, or variation in cerebral magnetic resonance imaging.
4. Any chronic non-psychiatric medical, neurological, or oncological illness.
5. No evaluable magnetic resonance imaging study.

### 2.2. Data Collection Tools and Procedure

The sociodemographic characteristics of the participants in the patient group, such as age and gender, and information on disease-related variables (smoking, alcohol and substance use history, family history of psychotic disorder, number of psychotic episodes, homicide and suicide attempt history, level of functionality determined by clinical history and treatment response evaluation, hospitalization) were retrospectively reviewed from the patient file archive.

Both radiologists involved in the study reached a consensus prior to the study on prototype cases (excluding the participants), taking into account the literature on measurement parameters, methodology, and reference points related to magnetic resonance images. The measurements of the participants’ magnetic resonance images were performed by a single radiologist who was blinded to age, gender, and clinical information.

### 2.3. Imaging and Measurement Methods

Brain magnetic resonance imaging (MRI) examinations of the groups were evaluated retrospectively through the image archiving and communication system (PACS). On T1-weighted sagittal axis images, the frontooccipital distance, the anterior–posterior length and area of the corpus callosum, and the thickness of the corpus callosum sections (rostrum, genu, body, isthmus, splenium) were measured by Witelson’s method, and the corpus callosum index was calculated.

***MRI protocol and imaging analysis:*** Imaging was performed with MRI devices with a 1.5 Tesla magnetic field strength and standard cranial MRI protocol using a standard head coil in the prone position. Sixty patients were imaged with GE Optima MR360 Advance (General Electric, Milwaukee, WI, USA), and 77 patients were imaged with Siemens Magnetom Aera (Siemens Medical Systems, Erlangen, Germany). It was ensured that the imaging of 137 patients in the control group was performed on the same MRI device as the corresponding patients in the patient group.

Morphometric measurements of the corpus callosum were performed on midsagittal axis T1-weighted (T1W) images, where the corpus callosum was best visualized. The parameters used in imaging were as follows: for GE: *repetition time* (TR): 511 ms; *echo time* (TE): 12 ms; slice thickness: 5.5 mm; slice gap: 1 mm; *field of view* (FOV): 260 mm; matrix: 288 × 192; *flip angle* (FA): 140°; bandwidth: 27.2 kHz; *number of excitations* (NEX): 1; for Siemens: TR: 319 ms; TE: 8.9 ms; slice thickness: 5.5 mm; slice gap: 1.7 mm; FOV: 255 mm; matrix: 256 × 205; FA: 90°; bandwidth: 149 Hz/Px; NEX: 1.

Images were evaluated using the ExtremePACS software program (version 3.4) installed on medical workstations. Brain images were realigned to standardize differences in head position tilt during image acquisition. The tilt in the sagittal axis was corrected by aligning the most anterior and most posterior points (ACC–PCC) of the corpus callosum (CC) so that the midsagittal axis passed through the same plane in the anterior to posterior direction and was parallel to the image plane.

**Figure 1:**
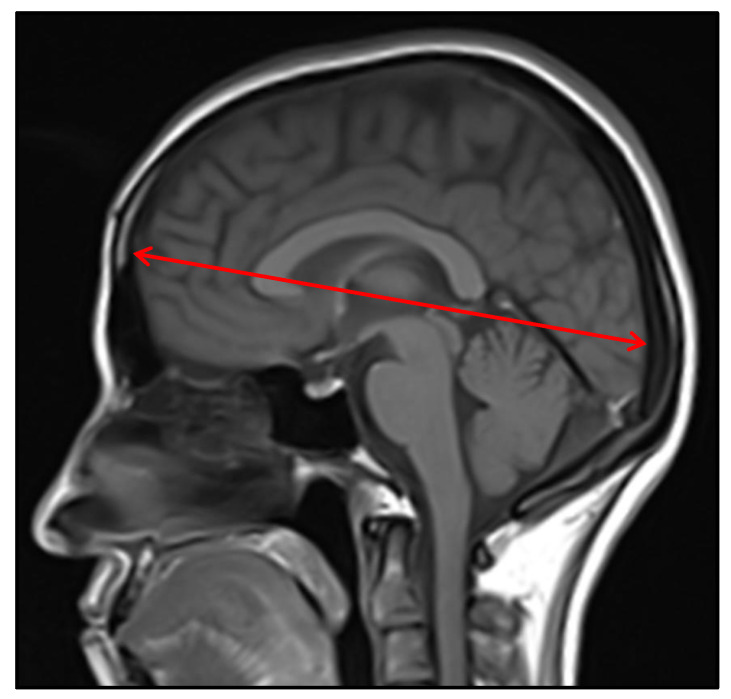
Fronto-occipital length measurement on midsagittal T1W MRI image.

**Figure 2:**
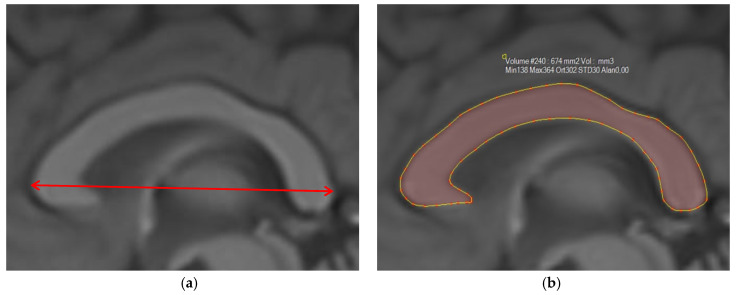
The midsagittal T1W MRI image shows (**a**) the anterior–posterior diameter and (**b**) the area of the corpus callosum.

The fronto-occipital length (Figure 1), defined as the length of the forebrain in the midsagittal plane, the AP diameter (Figure 2a), defined as the distance between the most anterior and most posterior points of the CC, and the CC area (Figure 2b), obtained by placing a *region of interest* (ROI) around the CC using the freehand method, were measured.

**Figure 3:**
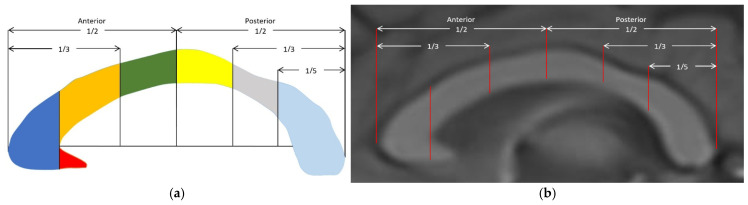
(**a**) Illustration and (**b**) midsagittal T1W MRI image of the corpus callosum segmented according to the Witelson method. Red: rostrum; dark blue: genu; orange: rostral body; green: anterior midbody; yellow: posterior midbody; gray: isthmus; light blue: splenium.

**Figure 4:**
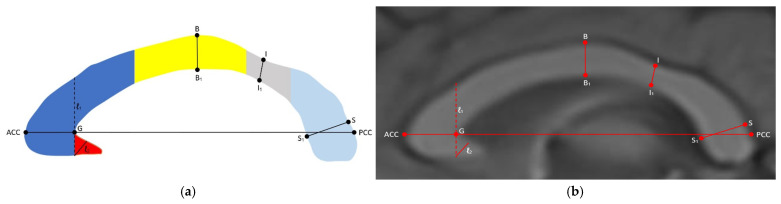
(**a**) Illustration and (**b**) midsagittal T1W MRI images of the corpus callosum segments and thicknesses. Red: rostrum (ℓ_2_); dark blue: genu (ACG–G); yellow: body (B–B_1_); gray: isthmus (I–I_1_); light blue: splenium (S–S_1_).

**Figure 5:**
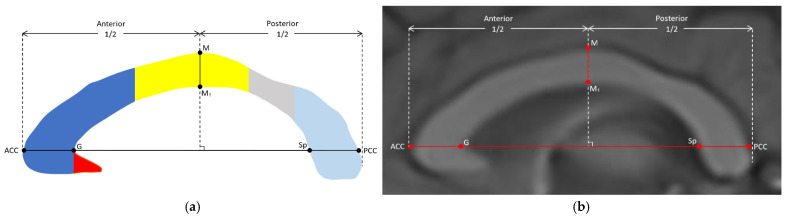
(**a**) Illustration and (**b**) midsagittal T1W MRI image of the segments of the corpus callosum and the thickness of the genu, splenium, and mid-callosal body in the widest part of the corpus callosum ([ACG–G], [Sp–PCC] and [M–M_1_], respectively). Red: rostrum; dark blue: genu; yellow: body; gray: isthmus; light blue: splenium. The corpus callosum index was calculated as the sum of these 3 values divided by the CC AP diameter (ACC–PCC).

The corpus callosum was segmented into the rostrum, genu, body, isthmus, and splenium according to the Witelson method, which is the most widely used method in the literature (Figure 3) [23]. According to the lines specified in this method, the genu and rostrum were separated by the craniocaudal virtual line (ℓ_1_) passing through the anterior inner convexity (G) of the CC. The thickness of the rostrum, which is a triangular structure, was measured with a perpendicular line (ℓ_2_) drawn from the lower anterior corner to the superomedial base of the rostrum. The thicknesses of the other segments were obtained by measuring the widest part of the length between the upper and lower edge of the CC perpendicular to the long axis of the CC for the genu, body, and splenium and the narrowest part for the isthmus (Figure 4). In the same image, the CC index was calculated by dividing the sum of the widest AP lengths between the anterior–posterior margin of the genu (ACC–G) and the anterior–posterior margin of the splenium (Sp–PCC) and the widest craniocaudal length (M–M_1_) between the superior and inferior margin of the body at the middle of the CC by the CC AP diameter (ACC–PCC) (Figure 5). The analysis of morphologic parameters is shown in detail in Table 1.

All length and thickness measurements were made in millimeters (mm), and area measurements were made in square millimeters (mm^2^). All measurements were performed by a radiology specialist with 12 years of experience who was blinded to age, gender, and clinical information. Measurements were conducted in a single session for each participant. To ensure the accuracy and reliability of the obtained values, the average of two measurements for each dimension and CC area was taken.

### 2.4. Statistical Analysis

In this study, the SPSS (Statistical Package for the Social Sciences) program version 22.0 (IBM Corp. Armonk, NY: USA) was used for statistical analysis of the data obtained from the participants. A descriptive statistical analysis of sociodemographic data was summarized as mean, standard deviation, median, number, and percentage. Chi-squared and Fisher’s exact tests were used to determine the relationship between categorical variables. Compliance with the normality assumption was tested with the Kolmogorov–Smirnov test. The Mann–Whitney U test was used for pairwise group comparisons of variables that did not meet the assumption, and the Student *t*-test was used for pairwise group comparisons of variables that met the assumption. In more than two group comparisons, an ANOVA was used for variables that met the normality assumption, and the Kruskal–Wallis test was used for variables that did not meet the assumption. In more than two group comparisons, Bonferroni and Tukey tests were used to determine from which group or groups the difference originated. The statistically significant difference level calculated for the study was accepted as *p* < 0.05.

## 3. Results

In our study, 274 individuals, including 137 schizophrenia patients (59 [43.1%] males; 78 [56.9%] females) and 137 healthy individuals (59 [43.1%] males; 78 [56.9%] females), were evaluated. There was no significant difference between the two groups in terms of age, gender, and mean age at the time of brain MRI (*p* ≥ 0.05). In the patient group, the mean age at diagnosis was 27.2 (±1.03) years and the mean disease duration was 13.5 (±0.9) years. The mean age of the patients at the time of MRI was 36.9 (±1.17) years and 36.9 (±13.6) years in the control group. There was no significant difference between the mean age at the time of MRI in the patient group and the healthy control group (*p* = 0.261).

In brain MRI measurements, forebrain length, CC AP diameter, CC genu, body, splenium, rostrum, and isthmus thickness, CC area, and CC index values were significantly lower in the patient group compared to healthy controls. A comparison of the morphologic measurements of the patient group and healthy control group is shown in Table 2.

The patient group was categorized according to the number of episodes they experienced since the onset of the disease. Accordingly, 98 (71.53%) of the patients had more than one episode (Group 1) and 39 (28.46%) had a single psychotic episode (Group 2). When the two groups were compared, the age at diagnosis was 24.7 (±9.5) in the group with multiple episodes and 33.5 (±15.2) in the group with a single episode, and the difference was statistically significant *(p* < 0.0001). In addition, the duration of the disease was 16.9 (±10.4) years in the group with multiple episodes and 4.87 (±5.05) years in the group with a single episode (*p* < 0.0001). There was no statistically significant difference between the mean age at MRI in both groups. In the group with multiple episodes, family history of psychotic disorder, history of homicide and suicide attempts, and number of hospitalizations were found to be higher, while the level of functionality was found to be lower in the group with multiple episodes compared to the group with single episodes. The characteristics of patients with single and multiple episodes are summarized in Table 3.

When the patient group and the healthy control group were compared in terms of forebrain length, CC subsegment thickness, CC area, and CC index measurements, all parameters were found to be significantly smaller in the patient group compared to the control group. When the subgroups were compared, a statistically significant difference was found only in the CC rostrum region between patients with a single episode and patients with multiple episodes (*p* = 0.048). In other areas, no statistically significant difference was found between patients with a single episode and those with multiple episodes. The forebrain length and CC measurements of the patient groups and healthy controls according to the number of episodes are shown in Table 4.

## 4. Discussion

Schizophrenia is a multifactorial chronic disease that causes disturbances in thought, perception, and affect and leads to destruction in intellectual and cognitive functions. Neurodevelopmental and neurodegenerative hypotheses have been proposed in its pathogenesis [24]. Brain morphological changes, which are thought to be important in the etiopathogenesis and course of the disease, can be used as a critical neuroimaging-based biomarker in understanding the neuroanatomical basis of schizophrenia and developing individualized treatment plans in clinical practice.

Research in the literature indicates that a significant proportion of schizophrenia patients experience multiple relapses, and each relapse is associated with adverse outcomes such as self-harm or harm to others, deterioration in interpersonal relationships, jeopardization of educational and occupational status, and increased stigmatization. Furthermore, it has been reported that relapses are also linked to the development of resistance to administered treatments and increased difficulty in returning to previous levels of functioning [25,26]. In light of all these data, we divided the patients into two groups, namely those who experienced a single episode and those with recurrent episodes. In our study, it was found that patients with multiple episodes had an earlier age at diagnosis and a longer duration of illness compared to patients with a single episode. The late onset of schizophrenia in single-episode patients could be explained by a combination of neurobiological resilience, environmental protection, higher cognitive reserve, better premorbid functioning, and reduced exposure to life stressors. In a systematic review and meta-analysis study in which 81 studies were evaluated, it was found that there was a statistically significant relationship between earlier age at onset and more hospitalizations, more relapses, worse social and occupational functioning, and worse overall outcomes in schizophrenia patients [27]. In our study, a history of homicidal–suicidal attempts, frequency of hospitalization, and positive family history of psychotic disorders were found to be statistically significantly higher in patients with multiple episodes compared to patients with a single episode. In the literature, recurrent relapses, earlier age at onset of the disease, decline in social and occupational functioning, treatment non-compliance, and a higher number of psychiatric hospitalizations were found to be associated with an increased risk of suicide in patients with schizophrenia [28,29,30,31,32,33]. In addition, various studies have shown that patients with an earlier age of onset have a higher family history of schizophrenia [34,35].

In the literature, there are many studies showing the relationship between the high number of relapses and poor functionality in patients with schizophrenia [36,37]. In our study, in line with the literature, the functioning level was significantly lower in the group with multiple episodes compared to the group with a single episode.

In schizophrenia patients, late age at onset of the disease was associated with a favorable prognosis, whereas recurrent episodes, a high number of hospitalizations, and a positive family history of schizophrenia were found to be indicators of an unfavorable prognosis [38]. Early onset of the disease is associated with recurrent episodes, frequent hospitalization, and impairment in functionality [38]. In our study, the age at onset of the disease was found to be relatively advanced in those who experienced a single episode; the good functionality in this group may be related to both the absence of relapse and late onset of the disease.

Suicide is another important problem in schizophrenia. Studies have reported that the rate of suicide attempts in schizophrenia patients in any period of their lives is between 18 and 55% [39,40,41]. In our study, the rate of suicide attempts was found to be significantly higher in patients with multiple episodes than in patients with a single episode. In the literature, factors that increase the risk of suicide in schizophrenia include early age of onset, the severity of the disease, a high number of relapses, and being single, unemployed, and lonely [28,29,30,31,32,33]. There may be various reasons why the rate of suicide attempts is lower in patients who have experienced a single episode and have a relatively later age of illness onset. Late onset of the disease is often linked to better premorbid functioning, including stable relationships, employment, and stronger social support networks. These protective factors may reduce the psychological burden and hopelessness often associated with early-onset cases, potentially lowering the suicide risk. Also, there may be differences in coping mechanisms. Individuals with a late onset might have more mature coping strategies, allowing them to better manage stress and psychotic symptoms without resorting to self-harm. Patients with multiple episodes often experience cumulative stress, leading to chronic feelings of hopelessness and despair. Repeated relapses can deteriorate social and functional outcomes, making patients more prone to suicidal ideation. In contrast, a single episode with long-term remission may instill a sense of recovery and hope, reducing suicide risk.

Various studies have suggested that symptoms of schizophrenia include the dysfunction of connections in cortical regions of the brain or dysfunction of the corpus callosum in information transmission in these regions; morphological changes in the shape and size of the corpus callosum may cause dysfunction by affecting the transmission and integration of information [42,43,44]. The corpus callosum (CC), whose primary task is inter-hemispheric information transmission, is also involved in the regulation of motor, sensory, and high-level cognitive signals [45]. While the posterior part of the CC is involved in visual, auditory, and somatosensory transmission, the anterior part is responsible for the integration of higher cognitive functions. According to the Witelson hypothesis, the larger size of the CC region is associated with a higher fiber number and myelination. It has been reported that the fiber number and myelinization density are related to functional or less functional hemispheric fibers passing through that region [23]. When we evaluated the brain morphometric measurements in our study, forebrain length, CC AP diameter, CC genu, body, splenium, rostrum, isthmus thickness, CC area, and CC index values were found to be significantly lower in the patient group compared to healthy controls. These findings are consistent with many studies in the literature [20,22,42,46,47,48].

In our study, only the CC rostrum thickness was found to be statistically significantly shorter in patients with schizophrenia with a single episode compared to those with multiple episodes. Other brain morphologic measurements were similar between the two groups. The similarity of other CC regions, apart from the rostrum, between patients with a single episode and those with multiple episodes may be interpreted as indicating a structural vulnerability associated with the disorder itself in schizophrenia patients. On the other hand, all of these measurements differed statistically significantly in the patient group compared to healthy controls.

Vermeulen et al. found the CC genu region to be larger in schizophrenia patients than in healthy controls [42]. However, many studies [20,46,49] have shown that the genu region of schizophrenia patients has a smaller area and thickness compared to healthy individuals. These studies emphasized that the reduction in the thickness and area of the genu may lead to impairments in information transfer due to changes in fiber integrity. In a study comparing first-episode and chronic schizophrenia patients with healthy controls, decreases in the thickness of the anterior genu region of the CC were found in first-episode schizophrenia patients, while similar decreases were found in the anterior genu, posterior genu, and isthmus regions in patients with chronic disease [46]. Downhill et al. [20] suggested that these changes in the genu region may negatively affect the information transfer between frontal association cortical regions, which may result in impairments in executive functions. In our study, the CC genu region was significantly smaller in the patient group compared to the healthy control group.

Various studies have shown decreased thickness and area of the CC anterior body and isthmus regions in schizophrenia patients [20,42,46]. The anterior body/posterior genu and isthmus regions connect the prefrontal, temporal, and inferior parietal cortical regions, which are compatible with those shown to be affected in schizophrenia patients. It is known that the impairments occurring in these regions may be associated with cognitive–executive dysfunction and positive psychotic symptoms in schizophrenia. In our study, consistent with the literature, these regions were found to be statistically significantly smaller in the patient group compared to healthy controls.

Francis et al. [47] and Prendergast et al. [48] reported that the posterior splenium region was smaller in schizophrenia and bipolar patients compared to healthy controls. In these studies, it has been suggested that smaller splenium regions may indicate disturbances in interhemispheric communication between the visual cortices and the prefrontal and temporoparietal association cortices responsible for executive functions. This may lead to cognitive dysfunctions and symptoms of schizophrenia patients [20,47]. In our study, the CC splenium region was found to be statistically significantly smaller in schizophrenia patients compared to healthy controls.

Another important finding of our study was that there was no significant size difference in CC subregions, except for the CC rostrum region when the groups with a single episode and those with multiple episodes were compared. This may be interpreted as supportive of the neurodevelopmental model suggesting that brain morphologic changes are present from the acute stages of the disease. This difference in the measurement of the rostrum region may be explained by brain plasticity, the activation of compensatory mechanisms, or the effects of the antipsychotic treatments used.

Arnone et al. [22], in their meta-analysis of 28 studies, reported that the CC area in the schizophrenia group was smaller than in the control group and was larger in chronic patients than in first-episode schizophrenia. Ahmadvand et al. [50] found that the CC area was smaller in schizophrenia patients compared to healthy controls. In another study in which schizophrenia patients were compared with healthy controls, no significant difference was found between the groups in terms of CC area [51]. Walterfang et al. showed a significant area reduction in the mid-anterior and posterior regions of the CC in patients with first-episode schizophrenia and an overall reduction in patients with chronic schizophrenia [52]. Colinson et al. [53] also reported a significant decrease in CC area in schizophrenia patients compared to the control group. In addition, the CC area of chronic schizophrenia patients was found to be larger compared to first-episode schizophrenia patients and the control group. In our study, the CC area was found to be statistically significantly smaller in schizophrenia patients compared to healthy controls.

Contradictory results were found in studies evaluating CC length in patients with schizophrenia. Some studies have found a decrease in CC length in patients with schizophrenia compared to healthy controls, while others have found an increase [54,55,56]. In our study, CC AP length was found to be lower in schizophrenia patients compared to healthy controls. Various studies have shown that callosal length and thickness may be significantly different depending on the age and gender of individuals [11,23]. The reason for these differences between studies may be related to variables such as the measurement methods used, age, gender, duration of the disease, number of episodes, and antipsychotic use. In our study, the fact that no significant difference was found between the mean age and gender of the patient and control groups at the time of MRI is important for the exclusion of age- and gender-related changes in brain morphology.

Studies have reported that in clinical practice, the measurement of the CC index has a strong correlation with other methods when compared to the measurements of the CC area and CC volume and is faster and more reliable than other methods [57,58]. Gonçalves et al. found a strong correlation between the CC index and whole brain volume [58]. Pérez-Martín et al. reported normative data on the CC index in adults [59]. Measurement of the CC index offers the opportunity to make individualized brain size corrections and obtain more accurate measurements [16]. In this context, it has been shown that it can be used as a reliable tool in the morphometric analysis of CC [16,58]. As far as we have seen, there is no study in the literature evaluating CC index measurements in schizophrenia patients. Our study found that CC index measurements were statistically significantly lower in schizophrenia patients compared to healthy controls, which may be interpreted as an indicator of decreases in the CC area and volume in schizophrenia patients. We believe that CC index measurement can be used in callosal measurements as an easily applicable and consistent method.

## 5. Conclusions

Our study found that corpus callosum subregion thickness and corpus callosum area and index measurements were each reduced in schizophrenia patients compared to healthy controls. Corpus callosum subregion thickness and corpus callosum area and index measurements show that schizophrenia patients have significant structural changes in these regions of the brain. These findings are important in understanding the neurobiological basis of the disease. However, the retrospective nature of our study is an important deficiency. Due to the retrospective design of our study and the inability to access information on the predominant symptoms supported by scales from each patient’s records, it was not possible to conduct this assessment. Additionally, another significant limitation of our study is that we were unable to address the treatment histories of the patients (such as medication, electroconvulsive therapy [ECT], transcranial magnetic stimulation [TMS], etc.) and their potential effects on the relevant measurements. Again, the fact that the relationship between clinical and neuropsychological tests and these anatomical regions was not examined in our study makes it difficult for us to interpret the study data.

The CC index, which offers the opportunity to make individualized brain size measurements, has not been previously evaluated in schizophrenia patients to the extent we have observed. This makes our study relevant. However, our results need to be confirmed by studies with a larger sample using a prospective study design in which clinical parameters related to the disease are also measured.

## Figures and Tables

**Table 1:** Morphological parameters of the corpus callosum evaluated in the study.

Parameter	Definition
Forebrain length (mm)	Distance between frontal pole and occipital pole
CC AP diameter (mm)	The distance between the most anterior point (genu, ACC) and the most posterior point (splenium, PCC) of the corpus callosum
CC segment thicknesses (mm)	The length between the upper and lower margins of the CC, perpendicular to the long axis of the CC, narrowest for the isthmus, and widest for the other segments
Rostrum	Perpendicular line drawn from the lower anterior corner of the rostrum to its superomedial base (ℓ_2_)
Genu	ACC–G
Body	B–B_1_
Isthmus	I–I_1_
Splenium	S–S_1_
CC index	[(ACC–G) + (Sp–PCC) + (M–M_1_)]/(ACC–PCC)
CC area (mm^2^)	The area within the line encircling the corpus callosum on the midsagittal axis

CC: Corpus callosum.

**Table 2:** Gender distribution and brain MRI measurements according to diagnosis groups.

	Patient Group (n = 137)		Control Group (n = 137)		*p*-Value
**Gender (n)**					1
Female	78 (56.9%)		78 (56.9%)		
Male	59 (43.1%)		59 (43.1%)		
**Forebrain length (mm)**	150.1 (±7.7)		154.6 (±6.7)		<0.0001 *
**CC AP diameter (mm)**	67.9 (±4.9)		68.9 (±4.2)		0.005 *
**CC genu (mm)**	10.6 (±1.6)		11.7 (±1.6)		<0.0001 *
**CC body (mm)**	5.7 (±0.8)		6.1 (±0.7)		<0.0001 *
**CC splenium (mm)**	10.8 (±1.6)		11.2 (±1.2)		0.014 *
**CC area (mm^2^)**	553.4 (±91.8)		591.6 (±78.2)		<0.0001 *
	**Mean rank**	**Sum of ranks**	**Mean rank**	**Sum of ranks**	
**CC rostrum**	116.83	16,005.5	158.17	21,669.5	**<0.0001** ^#^
**CC isthmus**	125.6	17,209	149.3	20,466	**0.013** ^#^
**CC index**	120.5	16,512.5	154.4	21,162.5	**<0.0001** ^#^

*: Student’s *t* test; ^#^: Mann–Whitney U test. CC: corpus callosum.

**Table 3:** Age of onset, duration of illness, and clinical variables according to the number of episodes.

	Group 1 Schizophrenia—Multiple Episodes (n = 98)	Group 2 Schizophrenia—Single Episode (n = 39)	*p*-Value
**Age of onset (years)**	24.7 (±9.5)	33.5 (±15.2)	**<0.0001**
**Duration of illness (years)**	16.9 (±10.4)	4.87 (±5.05)	**<0.0001**
**Smoking**			0.372 *
Yes	51 (52%)	17 (43.6%)	
No	47 (48%)	22 (56.4%)	
**Alcohol use**			0.774 ^#^
Yes	11 (11.2%)	5 (12.8%)	
No	87 (88.8%)	34 (87.2%)	
**Substance use**			0.062 ^#^
Yes	10 (10.2%)	0	
No	88 (89.8%)	39 (100%)	
**Family history of psychotic disorder**			**0.029** *
Yes	45 (45.9%)	10 (25.6%)	
No	53 (54.1%)	29 (74.4%)	
**Total number of episodes**			**<0.0001** ^#^
0–5 episodes	49 (50%)	39 (100%)	
6–10 episodes	41 (41.8%)	0	
>10 episodes	8 (8.2%)	0	
**History of homicide**			**0.009** ^#^
Yes	49 (50%)	10 (25.6%)	
No	49 (50%)	29 (74.4%)	
**History of suicide attempt**			**0.002** *
Yes	36 (36.7%)	4 (10.3%)	
No	62 (63.3%)	35 (89.7%)	
**Functionality level**			**<0.0001** *
Poor functionality	41 (41.8%)	2 (5.1%)	
Partially recovered	51 (52%)	15 (38.5%)	
Good functionality	6 (6.1%)	22 (56.4%)	
**Number of hospitalizations**			**<0.0001** ^#^
0–5 hospitalization	63 (62.4%)	38 (97.4%)	
6–10 hospitalization	30 (30.6%)	1 (2.6%)	
>10 hospitalization	5 (5.1%)	0	

*: Pearson chi-squared test; ^#^: Fisher’s exact test.

**Table 4:** Age at MR imaging, forebrain length, and corpus callosum measurements of patient groups and healthy controls according to the number of episodes.

	Group 1 Schizophrenia—Multiple Episodes (n = 98)	Group 2 Schizophrenia—Single Episode (n = 39)	Group 3 Control Group (n = 137)	*p*-Value
**Age at MR imaging (years)**	37.6 (±12.7)	35.1 (±15.8)	36.9 (±13.6)	0.261
**Forebrain length (mm)**	150.1 (±7.8)	150.09 (±7.5)	154.7 (±6.7)	**<0.0001**
**CC AP diameter (mm)**	68.3 (±5)	66.8 (±4.6)	68.9 (±4.2)	**0.033**
**CC rostrum (mm)**	3.7 (±0.8)	3.45 (±0.8)	3.9 (±0.6)	**<0.0001**
**CC genu (mm)**	10.6 (±1.5)	10.7 (±1.7)	11.7 (±1.6)	**<0.0001**
**CC body (mm)**	5.7 (±0.7)	5.6 (±0.9)	6.1 (±0.7)	**<0.000**
**CC isthmus (mm)**	3.8 (±0.7)	4.1 (±1.05)	4.1 (±0.7)	**0.003**
**CC splenium (mm)**	10.9 (±1.6)	10.4 (±1.3)	11.2 (±1.2)	**0.006**
**CC index**	0.402 (±0.045)	0.406 (±0.05)	0.42 (±0.03)	**0.001**
**CC area (mm^2^)**	562.5 (±88.7)	530.7 (±96.1)	591.6 (±78.2)	**0.027**

Mann–Whitney U and post hoc analysis. CC: corpus callosum.

## Data Availability

Research data are not publicly available but can be shared by the corresponding author upon request.
